# Elucidation of Nutritional Quality, Antinutrients, and Protein Digestibility of Dehulled and Malted Flours Produced from Three Varieties of Bambara Groundnut (*Vigna subterranean*)

**DOI:** 10.3390/foods14142450

**Published:** 2025-07-12

**Authors:** Mpho Edward Mashau, Thakhani Takalani, Oluwaseun Peter Bamidele, Shonisani Eugenia Ramashia

**Affiliations:** Department of Food Science and Technology, Faculty of Science, Engineering, and Agriculture, University of Venda, Thohoyandou 0950, South Africa; thakhani.takalani@univen.ac.za (T.T.); oluwaseun.bamidele@univen.ac.za (O.P.B.); shonisani.ramashia@univen.ac.za (S.E.R.)

**Keywords:** legume, germination, dehulling, nutritional composition, antinutrients, nutrient digestibility

## Abstract

Bambara groundnut (*Vigna subterranean*) is an important legume grain in sub-Saharan Africa, including South Africa. Nevertheless, the peculiarity of being hard to cook and mill and the availability of antinutritional factors often limit Bambara groundnut (BGN) use in food applications. This study investigated the impact of dehulling and malting on the nutritional composition, antinutritional factors, and protein digestibility of flours obtained from three BGN varieties (red, cream, and brown). Dehulling and malting significantly enhanced the moisture and protein content of BGN flours (dry basis), with values varying from 6.01% (control brown variety) to 8.71% (malted cream and brown varieties), and from 18.63% (control red variety) to 21.87% (dehulled brown), respectively. Dehulling increased the fat content from 5.82% (control red variety) to 7.84% (dehulled cream), whereas malting decreased the fat content. Nevertheless, malting significantly increased (*p* < 0.05) the fiber content from 4.78% (control cream) to 8.28% (malted brown variety), while dehulling decreased the fiber content. Both processing methods decreased the ash and carbohydrate contents of the BGN flours. Dehulling and malting significantly enhanced the amino acids of BGN flours, except for tryptophan and asparagine. Dehulling and malting notably increased the phosphorus, magnesium, potassium, and sulfur contents of the BGN flours, while calcium and zinc were reduced. Malting significantly enhanced the iron content of BGN flour, whereas dehulling reduced it. Both processing methods significantly enhanced palmitic, arachidic, and y-Linolenic acids. Nonetheless, processing methods significantly reduced phytic acid and oxalate, and dehulling achieved the most significant reductions. Dehulling and malting significantly enhanced the protein digestibility of the BGN flours from 69.38 (control red variety) to 83.29 g/100 g (dehulled cream variety). Overall, dehulling and malting enhanced the nutritional quality and decreased the antinutritional factors of BGN flours.

## 1. Introduction

Bambara groundnut (*Vigna subterranean* L.) is a legume belonging to the *Fabaceae* family and is native to Africa. It is a reasonable source of protein for consumers from poor households because they cannot afford protein from animal products [1]. Bambara groundnut (BGN) is rich in protein but also contains significant amounts of carbohydrates, dietary fiber, vitamins, and minerals. The protein content of BGN ranges from 20 to 25% and carbohydrate from 49 to 56%, which is similar to that found in other legumes, such as chickpea [2]. Bambara groundnut contains a good balance of essential amino acids, wherein lysine (8.5%) and methionine (6.4%) levels are higher [3,4]. The total dietary fiber in BGN varies from 1.4 to 10%, with insoluble fiber higher than soluble fiber. Bambara groundnut flour has more soluble fiber than other legumes [5]. Nonetheless, the maturity stage and processing methods influence the composition and amount of dietary fiber in BGN [6]. The major minerals in BGN are potassium, magnesium, phosphorus, zinc, and iron [7]. These minerals are higher than those in well-known legumes, including chickpeas and mung beans. Furthermore, BGN contains reasonable amounts of fatty acids such as palmitic acid (21%), oleic acid (23%), and linoleic acid (36%) [8].

Despite the demonstrated nutritional quality of BGN, its hard-to-cook and milling properties limit its use in food products. These phenomena develop during the storage of BGN at higher temperatures of up to 40 °C and high relative humidity above 75% [9,10]. Hard-to-cook BGN grains are normally boiled for a longer time (3–4 h), which results in high energy usage. This might pose a challenge to poor households that depend on firewood to supply energy to cook and heat BGN grains [10]. Nevertheless, the hard-to-cooking and milling phenomena of BGN grains are reduced by applying pretreatments, including soaking, dehulling, germination, malting, fermentation, and roasting before the grains are cooked or milled [11]. Mature BGN grains are consumed in different ways in southern Africa. For example, fresh-harvested pods are boiled for an hour and consumed as snacks. In Zambia, BGN grains are milled into flour and used to produce stiff porridge and baked bread [12]. Furthermore, in South Africa, BGN grains are cooked until soft and then pounded into a puree [6].

The usage and digestibility of BGN grains are limited by the presence of antinutritional factors (ANFs), such as phytic acid, oxalate, and tannin [13,14]. These compounds in BGN are believed to lower starch and protein digestibility, impair mineral absorption, and suppress the activity of proteolytic amylolytic enzymes [15]. Traditional techniques, including soaking, dehulling, and germination or malting, are utilized to reduce ANFs in legumes. Dehulling involves seed coat removal, in which antinutrients are concentrated. Previous studies have demonstrated that dehulling enhances the digestibility and taste of chickpea, pigeon-pea, and lentil and decreases the levels of phytochemicals or tannins, which are predominantly available in the hulls of legumes [16,17]. Oyeyinka et al. [6] reported the impact of soaking and dehulling on the proximate composition and ANFs of BGN flour. The results demonstrated that soaking and dehulling enhanced the protein and carbohydrate contents, whereas phytic acid and tannin contents were reduced.

Malting involves germination of grains in a controlled environment followed by drying [18]. During malting, starch is hydrolyzed into simpler sugars, and enzyme activity is enhanced [7]. Previous studies have reported the effect of germination on the nutritional composition, ANFs, and protein digestibility of BGN flours. Chinma et al. [1] germinated brown BGN up to 72 h. Results showed that germination enhanced the mineral, amino acid, and protein digestibility of brown BGN flour, whereas the ANFs were significantly reduced. Mudau and Adebo [19] determined the impact of malting on the proximate composition of mixed BGN flours. Malting significantly enhanced the protein, fat, and fiber content of BGN flour, whereas moisture, ash, and carbohydrate content decreased.

Bambara groundnut has recently garnered attention in South Africa because it is resistant to drought and has a high yield. The primary production regions of BGN in South Africa are the Limpopo, Mpumalanga, North-West, and KwaZulu-Natal provinces [20]. Seven BGN varieties are cultivated: cream/no eye, red, brown, black eye, speckled/flecked, and brown eye. Although BGN is gaining attention among small-scale farmers and rural growers, consumers are not aware of its production and processing, which eventually presents a challenge to its consumption [21]. South Africa, like other countries, is currently experiencing continuous drought, and the promotion of resilient and neglected crops such as BGN may be one strategy to reduce the impact of climate change [22]. In this context, information on the influence of dehulling and malting on the ANFs, amino acids, fatty acid profile, and protein digestibility of South African BGN varieties is scarce. Thus, this study aimed to evaluate the influence of dehulling and malting on the nutritional composition (proximate composition, amino acids, minerals, and fatty acids), ANFs (phytic acid and oxalate), and protein digestibility of three BGN varieties from South Africa. The findings of this study are anticipated to offer insight into the prospective use of BGN flour as a sustainable functional component in food applications.

## 2. Materials and Methods

### 2.1. Raw Materials and Chemicals

Mixed BGN grains (10 kg) were obtained from a supermarket in Thohoyandou, South Africa. The BGN grains used in this study were sorted into three varieties (red, brown, and cream). All the analytical chemicals and reagents were procured from Merck (Pty, Ltd., Midrand, Gauteng province, South Africa).

### 2.2. Production of Dehulled and Malted Bambara Groundnut Flour

All varieties of BGN were cleaned with tap water to remove dirt and infested grains. Each BGN variety was soaked separately in water for 20 h at an ambient temperature to facilitate dehulling. The water was thrown away, and the grains were manually dehulled via fingers sliding off the seed coat. Each dehulled BGN variety was placed on a tray and dried in a hot air oven (model 78, Labotech Ecotherm, Midrand, Gauteng province, South Africa) at 70 °C for 14 h to achieve a 12% moisture content or below [23]. The dried BGN grains were pulverized (Perten-3303 Laboratory Mill, Springfield, IL, USA) into flour and labelled as dehulled BGN flour.

For malting, the cream, brown, and red BGN grains (800 g) were steeped separately in water for 20 h at an ambient temperature (30 ± 2 °C). Soaking was performed with 8 L of distilled water using three 10 L round blue buckets. After steeping, the water was thrown away, and the grains were placed onto a tray and germinated at 28 °C for 120 h [24]. The grains were sprinkled with water twice daily to keep them wet during germination. Afterwards, the tray with malted BGN grains was dried in a convection oven at 70 °C for 14 h. Afterwards, the dried BGN grains were ground (Perten-3303 Laboratory Mill, Springfield, IL, USA) into flour and labelled as malted BGN flour. Whole BGN grains of each BGN variety (unprocessed) were ground into flour and labelled as control BGN flour. The control, dehulled, and malted flours of each BGN variety were placed in airtight plastic zip bags and stored at 4 °C until use.

### 2.3. Proximate Composition Analysis of Bambara Groundnut Flours

The moisture, ash, protein, fat, and crude fiber contents of the BGN flours were measured using the AOAC method [25]. The carbohydrate content was obtained by calculating the difference in percentage of moisture, fat, protein, ash, and crude fiber using the following formula: carbohydrates (%) = 100 − (moisture + ash + fat + protein + crude fiber).

### 2.4. Amino Acid Profile Analysis of Bambara Groundnut Flours

The individual amino acids of the BGN flours were quantified following the AOAC method [26], with minor alterations. Briefly, 500 mg of the BGN flour sample was hydrolyzed with 6 mL of 6 N hydrochloric acid (HCl) for 23 h at 110 °C. Subsequently, the mixture was allowed to cool to room temperature for 30 min. A Reacti-Therm™ heating/stirring module at 70 °C under a nitrogen stream was used to dry the mixture. The resulting supernatant was suspended in 100 mL volumetric flasks, and 0.1 M HCl was added to reach the final volume. The solution was filtered through A 0.22 μm pore size cellulose membrane syringe filters (Sigma–Aldrich, Johannesburg, Gauteng province, South Africa). The resultant residue was suspended in high-performance liquid chromatography vials (Quasar, PerkinElmer, Hopkinton, MA, USA). Orthophthalaldehyde and 9-fluorenylmethyl chloroformate were used to derivatize hydrolyzed samples. The sample was analyzed using a Zorbax Eclipse-AAA column at 40 °C for fluorescence detection.

The amino acid ratio of total essential amino acids (TEAA) to total non-essential amino acids (TNEAA) was calculated using the following formula:

TEAA: TNEEAA = TEAA/NEAA

### 2.5. Mineral Analysis of Bambara Groundnut Flours

The mineral content of the BGN flour samples was quantified using ICP emission spectroscopy (ICP-AES, Jarrel-Ash, Berkhamsted, Hertfordshire, UK ) following the procedure described by Mudau et al. [27]. Two grams of the BGN flour sample were burned to white ash using a muffle furnace for 3 h at 550 °C. Subsequently, 10 mL of hydrochloric acid solution and 5 mL of nitric oxide were combined with white ash. The mixture was placed in a water bath for 1 h, 10 mL of hydrochloric acid was added, and the mixture was suspended in a volumetric flask (100 mL). Distilled water was added to reach a volume of 100 mL in a volumetric flask. The flour sample was subjected to ICP-AES for mineral analysis, and the results were calculated as mg per 100 g.

### 2.6. Fatty Acid Profile Analysis of Bambara Groundnut Flours

The fatty acid profile of the BGN flours was measured using the method described by Choi et al. [28] with some modifications. Briefly, fatty acids (0.5 g) were extracted using n-hexane (2.5 mL) and 100 μL of an internal standard (heptadecanoic acid, 0.1 mL). To obtain the samples, 1 mL of 20% (*v*/*v*) methanolic sulfuric acid was added, mixed by vortexing, and incubated in a water bath for 1 h at 70 °C. Next, 2 mL of sodium chloride (20%, *w*/*v*) was added, and the mixture was kept at room temperature for 30 min. The solution was mixed by vortexing, and the top layer was suspended in clear 2 mL vials. The analysis and separation of fatty acid methyl esters (FAMEs) were performed using a gas chromatograph (GC, 6890 N, Agilent Technologies, Santa Clara, CA, USA) coupled to a flame ionization detector (FID, Palo Alto, CA, USA) with a polar RT-2560 (100 m, 0.25 mm ID, 0.20 μmol film thickness; Restek Corporation, Bellefonte, PA, USA) capillary column. The sample injection volume was 1 µL at a split ratio of 50:1. Helium was used as the carrier gas at a flow rate of 1.5 mL/min. A one μL of BGN flour sample was injected in a 5:1 split ratio, with the injector temperature held at 250 °C. The oven temperature was set at 100 °C and gradually increased to 170 °C at a rate of 60 °C/min, and with a holding time of 1 min. The temperature was then increased to 240 °C at 3 °C for 2 min. The analysis was performed using a mass spectrometer in electron impact mode at an ionization energy of 70 eV, scanning from 35 to 500 m/z. The fatty acid contents were reported as µg/g.

### 2.7. Antinutritional Factors Analysis of Bambara Groundnut Flours

The phytic acid content of the BGN flours was measured as described by Haji et al. [29]. Four grams of the BGN flour samples were steeped in 100 mL of 2% hydrochloric acid for 5 h and filtered. Subsequently, 25 mL of the filtrate was analyzed. The filtrate was then combined with 5 mL of 0.3% ammonium thiocyanate solution. An iron (III) chloride solution was used to titrate the mixture until a brownish-yellow color that persisted for 5 min was obtained.

The oxalate content in the BGN flours was evaluated using the titration method described by Agbaire [30]. To prepare 500 mL of 0.5 N H_2_SO_4_, concentrated sulfuric acid with 98% purity and a density of 1.84 g/mL was used. Concentrated acid (6.8 mL) was carefully measured and diluted with distilled water until the total volume reached 500 mL. To prepare 100 mL of 0.05 N potassium permanganate (KMnO_4_) solution, 0.158 g of potassium permanganate (KMnO_4_)_,_ with a molecular weight of 158 g/mol, was mixed with 100 mL of water. Briefly, a flour sample (0.5 g) was mixed with 75 mL of 3 M sulfuric acid and stirred for 60 min using a magnetic stirrer. Whatman filter paper no. 1 was used to filter the mixture. The filtrate (25 mL) was titrated while hot against 0.05 M potassium permanganate solution until a faint pink color persisted for at least 15–30 s, indicating the endpoint of the titration.

### 2.8. In Vitro Protein Digestibility Analysis of Bambara Groundnut Flours

The in vitro protein digestibility of the BGN flours was measured following the procedure described by Chinma et al. [31] with minor modifications. In brief, 500 mg of the flour sample was placed in a shaking water bath and digested with pepsin solution at 37 °C (pH 1.9 and 120 min). Subsequently, the samples were further digested with pancreatin solution for 120 min (pH 8.0). The digested samples were centrifuged at 4800 rpm at 4 °C for 20 min and filtered. The protein content of the residue was measured using the Dumas method. Protein digestibility was calculated as the difference between the percentage of undigested and hydrolyzed flour samples.

### 2.9. Statistical Analysis

The experiment was replicated, and the results were analyzed in triplicate. The analytical data are expressed as the mean ± standard deviation. The test data were analyzed by one-way analysis of variance (ANOVA) using SPSS software (version 27.0; SPSS Inc., Chicago, IL, USA). Significance was determined using Duncan’s multiple range test (*p* < 0.05).

## 3. Results and Discussion

### 3.1. Proximate Composition of Bambara Groundnut Flours

Table 1 depicts the proximate composition of the control, dehulled, and malted flours of the three BGN varieties. Dehulling and malting significantly enhanced (*p* < 0.05) the moisture content of all three varieties of BGN flours, with values varying from 6.01 (control brown) to 8.71% (malted cream and brown varieties). The malted red variety had the lowest moisture content (8.65%). For dehulled BGN flours, the red variety had the highest moisture content (7.82%), followed by brown (7.76%), and the cream had the lowest value of 7.42%. Regardless of the BGN variety, flours obtained from malted BGN had significantly higher moisture content compared to dehulled samples. The increased moisture content of the dehulled and malted BGN flours might be ascribed to the grains absorbing moisture during soaking and sprouting. It could be hypothesized that the grains underwent different metabolisms [32]. Nonetheless, the moisture content of the control, dehulled and malted BGN flour samples was within the safe moisture level of less than 10% for long-term storage of the flour. Similarly, Mao et al. [32] observed a rise in the moisture content of germinated chickpea flours.

Dehulling significantly increased (*p* < 0.05) the fat content of all BGN varieties, ranging from 6.56% (control cream variety) to 7.84% (dehulled cream variety). The dehulled brown and red varieties had a fat content of 7.17 and 7.16%, respectively. The fat content of dehulled cream, brown, and red varieties increased by 1.28, 1.23, and 1.34%, respectively. The increased fat content of dehulled BGN flours could be ascribed to seed coat removal and concentrated endosperm [33]. Similarly, Choi et al. [28] reported a decrease in the fat content of faba bean flour. This finding demonstrates that dehulled BGN flour can be used as a source of fat. Nonetheless, malting decreased the fat content of BGN varieties, with values varying from 6.56% in control cream to 5.12% in the malted red variety. The malted cream and brown varieties had a fat content of 5.21 and 5.14%, respectively. The fat content of malted cream, brown, and red varieties decreased by 1.35, 0.80, and 0.70%. The reduced fat content might be associated with the utilization of fatty acids as a source of energy by BGN grains during sprouting to carry out metabolic activities [34]. Fat is used to generate energy when lipase breaks down triglycerides to liberate free fatty acids. The reduced fat content of malted BGN flours is advantageous during storage because of the low levels of lipid oxidation [35]. Similarly, Lakshmipathy et al. [36] reported a rise in the fat content of dehulled grass pea flours.

Dehulling and malting significantly reduced the ash content of BGN varieties, with values varying from 3.08% (control red variety) to 2.33% (malted cream variety). The malted red and brown varieties had an ash content of 2.70 and 2.26%, respectively. For dehulled BGN flours, the red variety had the highest value (2.78%), followed by the cream (2.39%), and the brown variety had the lowest value (2.34%). The decreased ash content in the BGN flours could be ascribed to the leaching of minerals, especially water-soluble minerals, during soaking before dehulling and malting [37]. Nevertheless, the seed coats might have some minerals, and their removal might have resulted in the decreased ash content of dehulled BGN flours [38]. Similarly, Ferreira et al. [39] reported a decrease in the ash content of germinated chickpea flour, whereas Laksmiphathy et al. [36] reported a decrease in the ash content of dehulled grass pea flour. Contrarily, Atudorei et al. [35] observed an increase in ash content in germinated lentils, soybeans, and chickpea flours. The discrepancies observed in the results of these studies could be related to variations in the composition and functional properties of the flour used, primarily due to factors such as variety, growing conditions, and processing methods. Nonetheless, red BGN variety had higher ash content than the cream and brown varieties, and this might be ascribed to the increased activity of phytase, which might have degraded the bonds between the proteins, enzymes, and minerals, thereby releasing the minerals [35].

Dehulling and malting significantly enhanced (*p* < 0.05) the protein content of the BGN varieties, with values ranging from 18.63% (control red variety) to 21.87% (dehulled brown variety). The dehulled red and cream varieties had a protein content of 21.20 and 21.10%, respectively. For the malted BGN flours, the cream variety had a higher protein content (20.07%), followed by the brown variety (19.73%), and the red variety had a lower value of 19.53%. The increased protein content of dehulled BGN flours might be ascribed to the removal of the seed coats from the grains, thereby increasing the amount of endosperm [33,36]. The increased protein content of BGN flours during malting might be related to sprouting grains synthesizing enzymes, a change in the composition due to the disintegration of other components, and the synthesis of newly emerging proteins [40]. Furthermore, the activation of enzymes improves the metabolism and synthesis of proteins, leading to a higher amount of protein in malted grains [41]. Respiration during germination results in the loss of carbohydrates, thereby increasing the protein content of malted flours [42]. Similarly, Haji et al. [29] observed a rise in the protein content of germinated pigeon pea flours. The findings of this study showed that dehulled and malted BGN flours can be used as sources of protein.

Dehulling significantly reduced the crude fiber of the BGN varieties, with values ranging from 7.71% (control brown variety) to 3.26% (dehulled cream variety), respectively. Nevertheless, the dehulled brown and red varieties had fiber contents of 5.23 and 3.98%, respectively. Dietary fiber is the main constituent of the seed coat of legumes; its content is more than 70%, including less than 10% soluble fiber [43]. Therefore, removing the seed coats from the grains might have contributed to the decreased fiber content of the dehulled BGN flours. Nevertheless, the decreased fiber content of dehulled BGN flours might also be associated with leaching during soaking, which can contribute to the loss of soluble fiber constituents [29]. Similarly, Guajardo-Flores et al. [42] observed decreased fiber content in dehulled black beans. Malting significantly (*p* < 0.05) enhanced the crude fiber of BGN flours, ranging from 4.78% (control cream variety) to 8.28% (malted brown variety). The malted red variety had a fiber content of 6.95%, whereas the malted cream had the lowest value of 5.22%. The rise in fiber content of malted BGN flours could be linked to the synthesis of structural carbohydrates for cell wall formation [37]. Similarly, Moktan and Ojha [44] reported decreased fiber content in germinated horsegram flour. Previous studies by Soliman [45] and Li and Komarek [46] demonstrated that consuming food rich in dietary fiber has a protective effect against cardiovascular disease, diabetes mellitus, and cancer.

Dehulling and malting significantly reduced the carbohydrate content of the BGN varieties, with values varying from 60.86% (control cream variety) to 55.63% (dehulled brown variety). The seed coats of legumes contain fewer non-starch polysaccharides, including cellulose, hemicelluloses, and pectins, which contribute to dietary fiber. Thus, removing the seed coats during dehulling may have contributed to the decreased carbohydrate content in the BGN flours. Similarly, Lakshmipathy et al. [36] observed a reduction in the carbohydrate content of grass-pea flour. The reduced carbohydrate content of BGN flours during malting might be ascribed to the hydrolysis of carbohydrates into simple sugars by α-amylase enzymes, which improve digestibility [29]. Furthermore, α-amylase enzymes might disintegrate starch granules during malting, leading to reduced carbohydrate content in BGN flours [19]. Similarly, Haji et al. [29] reported a decreased carbohydrate content in germinated pigeonpea flour.

### 3.2. Amino Acid Profile of Bambara Groundnut Flours

Table 2 presents the amino acid (AA) profiles of the BGN varieties. Dehulling and malting significantly increased the levels of all essential AAs, except tryptophan, in all samples. Nonetheless, this effect was dependent on the BGN variety. For dehulled BGN flours, the greatest increase in AA was noted in leucine, ranging from 6.55 (control cream variety) to 12.21 mg/g (dehulled red variety), followed by phenylalanine, which ranged from 6.53 (control cream variety) to 9.33 mg/g (dehulled red variety). Malting significantly enhanced (*p* < 0.05) the leucine content of BGN flours, ranging from 6.55 (control cream) to 10.11 mg/g (malted brown variety), followed by phenylalanine, varying from 6.53 (control cream variety) to 7.85 mg/g (malted brown variety). Other essential AAs that increased after dehulling and malting included threonine, lysine, tryptophan (red variety), and others. Furthermore, both processing methods enhanced methionine, which is a limiting AA in legume flours such as BGN, varying from 0.94 (control cream variety) to 1.67 mg/g (dehulled brown variety) and 1.44 mg/g in malted brown variety. Dehulling and malting significantly decreased the tryptophan of cream and brown varieties of BGN flours, with values varying from 3.05 (control cream variety) to 2.19 mg/g (malted brown variety). Tryptophan serves as a precursor for melatonin biosynthesis during germination, and its decrease may be related to this action. Nonetheless, the tryptophan content of malted red BGN flour was higher than that of control BGN flour. The rise in tryptophan might be related to the activation of new metabolic pathways during malting because of the degradation of storage proteins [47].

Dehulling and malting significantly enhanced the non-essential AAs with glutamic acid having the greatest increase ranging from 27.10 (control cream variety) to 33.24 mg/g (dehulled red variety) and 30.19 mg/g (malted brown variety), followed by aspartic acid, which ranged from 17.84 (control red variety) to 24.04 mg/g (dehulled red variety) and 22.36 mg/g (malted brown variety). Alanine, glycine, and proline were other non-essential AAs that increased after dehulling and malting. Asparagine was not affected by the processing methods, with values varying from 0.10 (control red and dehulled brown varieties) to 0.12 mg/g (dehulled and malted red varieties). The increase in essential and non-essential AAs in the malted BGN flours might be linked to the degradation of storage proteins and activation of metabolic pathways such as shikimic acid during malting [47]. The shikimic pathway promotes the de novo synthesis of AAs, especially aromatic acids such as phenylalanine and tyrosine [48]. Moreover, malting may induce protein reserves in the cotyledons associated with synthesizing new proteins for sprouted grains’ growth. The new protein might consist of different AAs than storage proteins, thus modifying the pattern of AA content [49].

Nevertheless, the decrease in cysteine in the cream variety of BGN flour might be related to its usage as a source of energy during the early stages of malting. This might have led to cysteine not being enhanced during enzymatic hydrolysis during malting. The increase in aromatic AAs, including phenylalanine and tyrosine, after malting is beneficial because they are needed to synthesize secondary metabolites such as phenolic acids and lignin [50]. The high amounts of valine and leucine in dehulled and malted BGN flours are advantageous because they are vital for the division of cells, distribution of carbon, and metabolism of nitrogen during the sprouting of BGN grains [51]. The increased essential and non-essential AAs of dehulled BGN flours demonstrate that AAss are present in the greatest amount in cotyledons. Similarly, Chinma et al. [1] and Díaz-Batalla et al. [47] reported an increased AA profile of brown germinated BGN and common bean flours.

Furthermore, the high levels of glutamic acid and arginine in dehulled and malted BGN flours play an important role in radicle elongation and cell division [52]. The increment in the methionine content of processed BGN flours is beneficial because it is necessary to maintain the tissue protein. The high amounts of leucine, isoleucine, and valine in dehulled and malted BGN flours are advantageous because they are essential for building muscle tissues [50]. The increased glutamic acid content of processed BGN flours is vital because it acts as a substrate for proline and arginine, which are involved in grain respiration [53,54]. High levels of glutamic acid are also associated with anti-diabetic properties, whereas AAs such as lysine, isoleucine, alanine, and arginine improve the secretion of insulin [55]. Arginine is required for the synthesis of other AAs [56]. Nevertheless, arginine and histidine are necessary for the growth and development of young children. The results showed that processed BGN flour is a rich source of AAs and may be utilized as a functional component in food products.

As shown in Table 2, the BGN flours had an AA ratio of 0.41 of total essential AAs to total non-essential AAs. This ratio (0.41) indicates a low-quality protein source, which may be insufficient as a sole dietary protein. The ratio is below the recommended ratio of approximately 0.6 (FAO/WHO standard for good-quality protein). A low ratio suggests that the protein may not support optimal human growth or maintenance, especially in populations with higher protein needs, such as young children and pregnant women. Therefore, BGN flours need to be supplemented or combined with other protein sources richer in essential AAs to meet nutritional requirements. Essential AAs are not produced by the body, unlike the non-essential AAs; they must be obtained from the diet. Essential AAs are crucial for protein synthesis and support muscle growth and repair [57]. Furthermore, they regulate the breakdown of muscle protein, thereby corroborating a balance between protein synthesis and breakdown [58].

### 3.3. Mineral Content of Bambara Groundnut Flours

The mineral contents of the BGN flours are listed in Table 3. The calcium and zinc levels of the dehulled and malted BGN flours were lower than those of the control BGN flours, except for the calcium of the control and dehulled cream variety, which showed no significant difference. The values of calcium in dehulled BGN flours ranged from 0.94% (red variety) to 0.77% (dehulled brown), while for malted BGN flours, they varied from 0.91% (red variety) to 0.82% (cream variety). The values of zinc in dehulled BGN flours varied from 0.98% (red variety) to 0.77% (brown variety), whereas they varied from 0.91% (red variety) to 0.82% (cream and brown varieties). Dehulling significantly reduced the iron content of BGN flours, with values ranging from 0.71 (red variety) to 0.54% (cream variety), respectively. The decreased calcium, zinc, and iron content in the processed BGN flours could be related to the leaching of these minerals during soaking before dehulling and malting. Furthermore, Bains et al. [59] reported that longer germination times resulted in decreased mineral content. The reduced calcium, zinc, and iron contents of dehulled BGN flours might be ascribed to the removal of the seed coats, thereby leaving the cotyledons with a minimal amount of calcium and zinc. Yahaya et al. [23] and Haji et al. [29] observed similar decreases in calcium content in dehulled BGN flours and reduced iron content in germinated pigeon pea flour.

However, contrary results of increased calcium content in germinated brown BGN and pigeon pea flours were reported by Chinma et al. [1] and Haji et al. [29]. These disparities in the findings may be related to the synthesis of calcium during germination. Dehulling and malting increased the phosphorus, magnesium, potassium, and sulfur content of the BGN flours. The values of phosphorus ranged from 49.48% (control red variety) to 55.82% (malted cream), from 2.53% (control cream variety) to 4.65% (malted cream) for magnesium, respectively. The values of potassium varied from 0.35 (control red variety) to 1.44% (malted brown variety), whereas sulfur ranged from 49.46 (red control variety) to 55.87% (malted cream variety).

**Figure 1:**
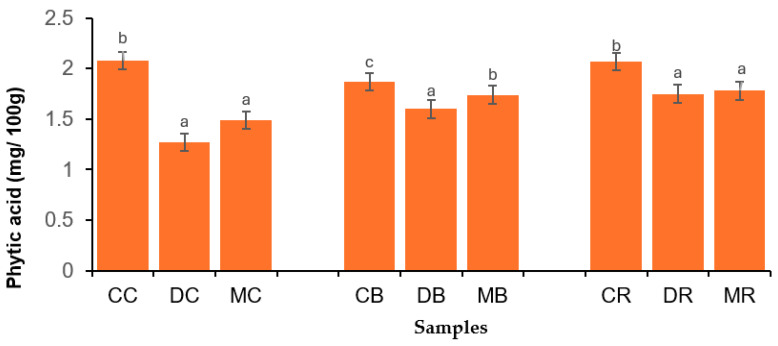
Phytic acid of Bambara groundnut flours. CC = control cream, DC = dehulled cream, MC = malted cream, CB = control brown, DB = dehulled brown, MB = malted brown, CR = control red, DR = dehulled red, and MR = malted red varieties. Error bars with different superscripts indicate that the mean values are significantly different (*p* < 0.05).

**Figure 2:**
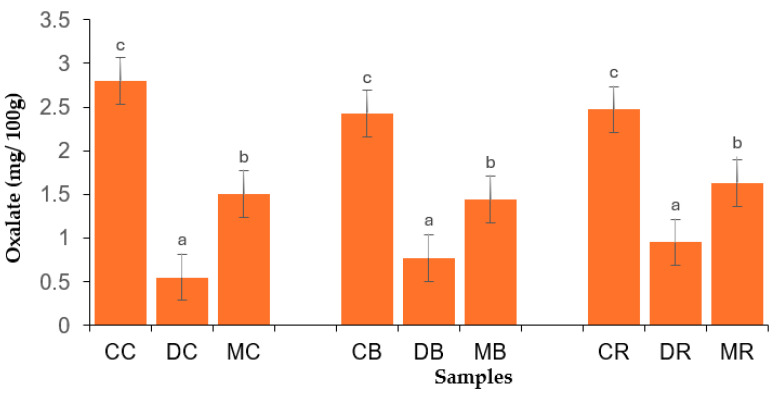
Oxalate content of Bambara groundnut flour samples. CC = control cream, DC = dehulled cream, MC = malted cream, CB = control brown, DB = dehulled brown, MB = malted brown, CR = control red, DR = dehulled red, and MR = malted red varieties. Error bars with different superscripts indicate that the mean values are significantly different (*p* < 0.05).

Malting significantly enhanced the iron content of BGN varieties, with values ranging from 0.98 (red variety) to 1.12% (brown variety). The increased mineral content of dehulled and malted BGN flours might be related to the lower levels of ANFs, as shown in Figure 1 and Figure 2. Furthermore, bonds and complexes may have been broken down during dehulling and malting to release minerals that were confined by ANFs, which affected their bioavailability [60]. Similarly, Elebouike et al. [61] reported an increase in magnesium, phosphorus, and potassium contents in sprouted mungbean flour. In summary, dehulling and malting significantly enhanced the major minerals (phosphorus, magnesium, potassium, and sulfur), whereas calcium was reduced. Furthermore, dehulling significantly reduced the zinc (cream and brown varieties) and iron of the BGN varieties. On the other hand, malting significantly reduced the zinc of BGN varieties, while iron was enhanced. The high amounts of minerals in processed BGN flours are useful for human health because they play important roles in building strong bones and teeth, maintaining fluid balance, and converting food into energy [62].

### 3.4. Fatty Acid Composition of Bambara Groundnut Flours

The fatty acid composition of the BGN flour is presented in Table 4. Dehulling and malting significantly (*p* < 0.05) increased the levels of saturated fatty acids, including palmitic and arachidic acids, but this was dependent on the BGN variety. The greatest increase was observed in palmitic acid of cream variety, with values ranging from 235.19 (control) to 242.61 µg/g (dehulled and malted), followed by arachidic acid of cream variety, ranging from 23.01 (control) to 23.47 µg/g (malted). Furthermore, dehulling and malting significantly enhanced unsaturated fatty acids, such as y-Linolenic of all BGN varieties, elaidic (cream and brown), linolelaidic, and α-Linolenic (cream variety). The greatest increase was noted in linolelaidic acid of cream variety, ranging from 440.46 (control) to 444.52 µg/g (dehulled), followed by elaidic acid of brown variety, ranging from 278.89 (control) to 280.28 µg/g (malted). Nevertheless, α-Linolenic acid of the cream variety ranged from 27.77 (control) to 29.52 µg/g (malted).

In general, palmitic acid was the dominant saturated fatty acid in BGN flour, ranging from 235.19 (control cream variety) to 242.61 µg/g (dehulled and malted cream variety). Stearic acid was the second dominant saturated fatty acid, ranging from 74.64 (control red variety) to 78.35 µg/g (control cream variety), and behenic acid varied from 72.96 (control cream variety) to 74.10 µg/g (dehulled cream variety). Other notable saturated fatty acids include arachidic, myristic, and margaric fatty acids. The minor saturated fatty acid in BGN flours was palmitoleic, ranging from 2.00 (control red variety) to 2.05 µg/g (control cream and dehulled brown varieties). The dominant unsaturated fatty acid was linolelaidic acid, ranging from 440.46 (control cream) to 447.12 µg/g (control red variety), followed by elaidic acid, varying from 272.10 (control cream variety) to 287.80 µg/g (control red variety), and α-Linolenic acid, ranging from 27.77 (control cream variety) to 30.38 µg/g (control red variety). Other notable unsaturated fatty acids include y-Linolenic, docosadienoic, and eicosenoic acids. The minor unsaturated acid in BGN flours was Dihomo-γ-linolenic, ranging from 4.02 (control brown) to 4.37 µg/g (control red variety). The high amounts of unsaturated fatty acids make processed BGN an ideal legume for utilization as a functional ingredient in food applications.

The increased amounts of these saturated and unsaturated fatty acids in dehulled BGN flours show that they are more available in the cotyledons than in the seed coat (hull). Contrarily, Pal et al. [16] reported a decreased palmitic acid content in dehulled lentil flour. Nevertheless, dehulling did not affect the linolenic acid content of the lentil flour. The enhancement of saturated and unsaturated fatty acids in malted BGN flours might be related to free fatty acids not being converted to carbohydrates during malting [63]. Similarly, Pal et al. [16] reported an increase in linolenic acid content in germinated lentil flours. Diets high in omega-3 fatty acids (mainly α-linolenic acid) have been associated with a reduced incidence of cardiovascular disease, whereas γ-linolenic acid (omega-6 fatty acid) is involved in the synthesis of other fatty acids such as arachidonic acid [64]. Despite such benefits, the presence of high amounts of unsaturated fatty acids has a negative influence on the shelf life and stability of BGN oils because they are vulnerable to oxidation [65]. Dehulling and malting reduced the saturated fatty acid such as palmitic of brown variety from 243.99 (control) to 242.34 µg/g (dehulled) and from 248.11 (control red variety) to 242.41 µg/g (malted red variety), stearic acid of brown variety from 78.35 (control) to 75.95 (malted), and margaric of red variety (2.98, control to 2.93 µg/g, dehulled, and malted), respectively.

The myristic, palmitoleic, and behenic acids of all BGN varieties were not affected by dehulling or malting. Margaric acid in cream and brown varieties, stearic and arachidic acids in brown and red varieties, and palmitoleic acid in cream and red varieties were also not affected by processing methods. Furthermore, dehulling and malting reduced unsaturated fatty acids such as eicosenoic, docosadienoic, and dihomo-y-linolenic acid. Linoleic and α-linolenic acids of brown and red varieties were also unaffected by the processing methods. The reduction in saturated and unsaturated fatty acids in dehulled BGN flours indicated that these fatty acids were abundant in the seed coat. Furthermore, dehulling is believed to influence the degradation of fats, thereby exposing legumes to fatty acids [66]. In contrast, Pal et al. [16] reported a reduction in stearic acid in dehulled lentil flour, whereas germination did not show any significant variation. The reduction in fatty acids in malted BGN flours could be associated with their hydrolysis to produce the energy needed for biochemical processes [67]. Nevertheless, few studies have investigated the impact of dehulling on the individual fatty acid profiles of legumes [68]. In this context, this is the first study to evaluate the influence of dehulling and malting on individual fatty acids in BGN flours of different varieties.

### 3.5. Antinutritional Factors of Bambara Groundnut Flours

Anti-nutritional factors (ANF) were assessed by phytic acid and oxalate content (Figure 1 and Figure 2, respectively. Dehulling and malting significantly decreased the phytic acid of BGN flours, with values ranging from 2.08 to 1.49 mg/100 g (cream variety), 1.87 to 1.74 mg/100 g (brown variety), and from 2.07 to 1.78 mg/100 g (red variety), respectively. Furthermore, the oxalate in BGN flours was reduced by processing methods, with values ranging from 2.80 to 0.55 mg/100 g (cream variety), from 2.43 to 0.77 mg/100 g (brown variety), and from 2.47 to 0.95 mg/100 g. Phytic acid is found at higher levels in the hulls and germ of plant seeds [69]; thus, the removal of the hull (seed coat) might have contributed to the decreased phytic acid in dehulled BGN flours. Dehulling reduces ANFs that interfere with protein digestibility because polyphenols, especially those of high molecular weight, can precipitate proteins and decrease protein digestibility [70].

The malting process enhances the activity of the phytase enzyme, which hydrolyzes phytate phosphorus into inositol monophosphate, thereby decreasing the phytic acid in malted BGN flours [37]. The phytase enzyme makes phytates soluble, thereby releasing soluble proteins and minerals. High amounts of phytic acid are not beneficial because they decrease the availability of macrominerals, whereas phytate phosphorus is not available to humans [16,38]. Similarly, Haji et al. [29] reported a reduced phytic acid content in germinated pigeon pea flour. Bambara groundnut grains were soaked in water before dehulling and malting. Thus, phytic acid and oxalate, which are water soluble, might have leached out, thereby contributing to their decreased values in the dehulled and malted BGN flours. The decreased oxalic acid in malted BGN flours might be associated with the oxalate oxidase enzyme, which might have cleaved oxalic acid, resulting in further leaching during soaking [71]. Furthermore, the same enzyme might have disintegrated oxalic acid into carbon dioxide and hydrogen peroxide, resulting in a decrease in malted BGN flours [72]. Similarly, Pal et al. [33] observed a decrease in oxalic acid in germinated horsegram flours. The reduced phytic acid and oxalate contents in this study corroborated enhanced protein content, amino acids, minerals, and protein digestibility.

### 3.6. Protein Digestibility of Bambara Groundnut Flours

**Figure 3:**
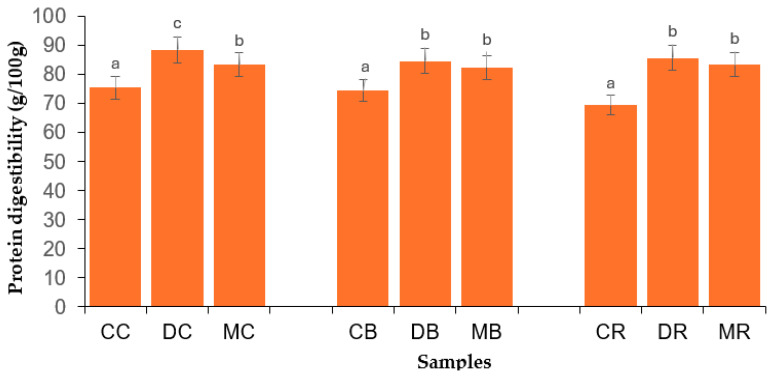
Protein digestibility of Bambara groundnut flour samples. CC = control cream, DC = dehulled cream, MC = malted cream, CB = control brown, DB = dehulled brown, MB = malted brown, CR = control red, DR = dehulled red, and MR = malted red varieties. Error bars with different superscripts indicate that the mean values are significantly different (*p* < 0.05).

Figure 3 shows the protein digestibility of the BGN flours. Dehulling and malting significantly enhanced the protein digestibility of BGN flour. The values varied from 75.30 to 83.29 g/100 g (cream variety), 74.37 to 82.35 g/100 g (brown variety), and from 69.38 to 83.20 g/100 g (red variety). The increased protein digestibility of dehulled BGN flours might be linked to the removal of polyphenols present in seed coats (hulls) during dehulling [42]. Mang et al. [73] indicated that the cotyledons of seeds have low amounts of polyphenols and are abundant in the seed coats. Furthermore, lower levels of phytic acid and oxalate (Figure 1 and Figure 2, respectively) might be ascribed to the enhanced protein digestibility of the dehulled BGN flours. Nevertheless, ANFs are not the only factors responsible for decreasing protein digestibility. For example, dietary fiber may decrease protein digestibility, but the rise in soluble dietary fiber and reduced insoluble dietary fiber during processing might be associated with the enhancement of protein digestibility [74]. Thus, the enhancement of protein digestibility might be associated with a decreased crude fiber content of dehulled BGN flours, as shown in Table 1.

The increased protein digestibility of malted BGN flours might be related to the enhanced activity of endogenous enzymes, which might have degraded large protein molecules into smaller ones [41,75]. Furthermore, sprouting might have caused the mobilization of proteins assisted by proteases, which led to the production of peptides, oligopeptides, and free amino acids, thereby increasing protein digestibility [76]. Nonetheless, the increased protein digestibility of the malted BGN flours may be related to the low levels of ANFs. Phytic acid and polyphenols interact with proteins to form complexes. Thus, these interchanges might result in the increment of the cross-linking degree, reducing the solubility of proteins and producing complexes of proteins that weaken the access of proteases to volatile peptide bonds [74].

The high protein digestibility of processed (dehulled and malted) BGN flours is beneficial because the body has a better chance of absorbing and using more protein to support tissue repair, muscle growth, and overall health [77]. Furthermore, both processing methods can be used to obtain high-quality proteins from BGN flour, which may be utilized as functional ingredients in food applications.

## 4. Conclusions

The results of this study demonstrate that dehulling and malting significantly enhance the protein content, amino acids, major minerals, and protein digestibility of BGN flours. Nevertheless, both processing methods reduce the ash, carbohydrate content, and calcium. Dehulling significantly reduced the zinc (cream and brown varieties) and iron of the BGN varieties. Nevertheless, malting significantly reduced the zinc of BGN varieties, while iron was increased. Dehulling increases the fat content, whereas malting enhances the fiber content of BGN flour. This shows the potential of dehulling and malting to enhance the nutritional quality of BGN flours. Moreover, both processing methods reduced antinutritional factors (phytic acid and oxalate). The reduction in antinutritional factors in BGN flour is important because they interfere with the digestibility and accessibility of nutrients. This involves dehulling and malting as auspicious processing methods to improve the utilization of BGN flours in different food applications. Based on the results of this study, future research should focus on developing functional foods from dehulled and malted BGN flour.

## Figures and Tables

**Table 1:** Proximate composition of control dehulled and malted Bambara groundnut flours (dry basis).

Samples	Moisture (%)	Fat (%)	Ash (%)	Protein (%)	Crude Fiber (%)	Carbohydrates (%)
Cream Control	6.55 ± 0.03 ^a^	6.56 ± 0.03 ^b^	2.88 ± 0.16 ^b^	19.37 ± 0.06 ^a^	4.78 ± 0.17 ^b^	60.86 ± 0.23 ^c^
Dehulled	7.42 ± 0.04 ^b^	7.84 ± 0.10 ^c^	2.39 ± 0.01 ^a^	21.10 ± 0.17 ^c^	3.26 ± 0.16 ^a^	57.99 ± 0.18 ^a^
Malted	8.71 ± 0.09 ^c^	5.21 ± 0.03 ^a^	2.33 ± 0.27 ^a^	20.07 ± 0.06 ^b^	5.22 ± 0.18 ^c^	58.46 ± 0.40 ^b^
Brown Control	6.01 ± 0.06 ^a^	5.94 ± 0.04 ^b^	2.98 ± 0.12 ^c^	18.97 ± 0.06 ^a^	7.71 ± 0.26 ^b^	58.39 ± 0.23 ^b^
Dehulled	7.76 ± 0.07 ^b^	7.17 ± 0.04 ^c^	2.34 ± 0.01 ^b^	21.87 ± 0.06 ^c^	5.23 ± 0.35 ^a^	55.63 ± 0.12 ^a^
Malted	8.71 ± 0.20 ^c^	5.14 ± 0.03 ^a^	2.26 ± 0.13 ^a^	19.73 ± 0.12 ^b^	8.28 ± 0.66 ^c^	55.88 ± 0.21 ^a^
Red Control	6.20 ± 0.12 ^a^	5.82 ± 0.04 ^b^	3.08 ± 0.04 ^b^	18.63 ± 0.06 ^a^	5.93 ± 0.47 ^b^	60.34 ± 0.13 ^c^
Dehulled	7.82 ± 0.01 ^b^	7.16 ± 0.03 ^c^	2.78 ± 0.12 ^a^	21.20 ± 0.00 ^c^	3.98 ± 0.29 ^a^	57.06 ± 0.08 ^a^
Malted	8.65 ± 0.05 ^c^	5.12 ± 0.08 ^a^	2.70 ± 0.07 ^a^	19.53 ± 0.15 ^b^	6.95 ± 0.24 ^c^	57.68 ± 0.27 ^b^

The results are reported as the mean ± standard deviation of three replicates. Mean values in columns with different letters differ significantly (*p* ≤ 0.05).

**Table 2:** Amino acid profile of control dehulled and malted Bambara groundnut flours (mg/g).

**BGN Variety**	**Essential Amino Acids**									
**Samples**	**Valine**	**Leucine**	**Isoleucine**	**Methionine**	**Threonine**	**Phenylalanine**	**Lysine**	**Histidine**	**Tryptophan**	
Cream Control	2.68 ± 0.54 ^a^	6.55 ± 1.13 ^a^	1.84 ± 0.39 ^a^	0.94 ± 0.07 ^a^	4.80 ± 0.62 ^a^	6.53 ± 0.43 ^a^	4.41 ± 0.45 ^a^	2.18 ± 0.01 ^a^	3.05 ± 0.30 ^b^	
Dehulled	4.22 ± 0.95 ^b^	9.79 ± 1.18 ^c^	2.95 ± 0.76 ^b^	1.28 ± 0.23 ^b^	5.86 ± 0.36 ^b^	8.59 ± 0.62 ^c^	5.84 ± 0.17 ^c^	2.35 ± 0.26 ^b^	2.58 ± 0.37 ^a^	
Malted	3.96 ± 0.73 ^b^	8.93 ± 1.36 ^b^	2.64 ± 0.42 ^b^	1.33 ± 0.02 ^b^	5.51 ± 0.52 ^b^	7.17 ± 0.45 ^b^	4.98 ± 0.18 ^b^	2.66 ± 0.18 ^c^	2.53 ± 0.27 ^a^	
Brown Control	3.73 ± 0.36 ^a^	8.45 ± 0.87 ^a^	2.55 ± 0.25 ^a^	1.33 ± 0.06 ^a^	4.76 ± 0.11 ^a^	7.16 ± 0.14 ^a^	5.20 ± 0.29 ^a^	2.39 ± 0.22 ^a^	3.02 ± 0.11 ^c^	
Dehulled	4.22 ± 0.54 ^b^	9.61 ± 0.44 ^b^	2.95 ± 0.35 ^b^	1.67 ± 0.15 ^c^	6.36 ± 1.00 ^b^	8.89 ± 1.13 ^b^	6.04 ± 0.36 ^b^	2.39 ± 0.03 ^a^	2.68 ± 0.16 ^b^	
Malted	4.56 ± 0.44 ^b^	10.11 ± 0.69 ^b^	3.01 ± 0.23 ^b^	1.44 ± 0.06 ^b^	6.29 ± 0.45 ^b^	7.85 ± 0.35 ^b^	5.73 ± 0.25 ^b^	2.57 ± 0.05 ^b^	2.19 ± 0.04 ^a^	
Red Control	2.88 ± 0.29 ^a^	6.94 ± 0.57 ^a^	1.91 ± 0.18 ^a^	0.97 ± 0.08 ^a^	5.09 ± 0.12 ^a^	6.12 ± 0.17 ^a^	4.27 ± 0.61 ^a^	2.07 ± 0.01 ^a^	3.42 ± 0.17 ^b^	
Dehulled	5.46 ± 0.75 ^c^	12.21 ± 2.08 ^c^	3.87 ± 0.76 ^c^	1.35 ± 0.15 ^b^	6.30 ± 0.17 ^b^	9.33 ± 1.89 ^c^	6.29 ± 0.59 ^c^	2.81 ± 0.58 ^b^	2.32 ± 0.00 ^a^	
Malted	3.48 ± 0.05 ^b^	8.13 ± 0.23 ^b^	2.34 ± 0.05 ^b^	1.21 ± 0.02 ^b^	6.16 ± 0.31 ^b^	7.09 ± 0.39 ^b^	5.24 ± 0.73 ^b^	2.57 ± 0.33 ^b^	3.86 ± 0.47 ^c^	
Total EAA	35	81	24	12	51	69	48	20	24 = 364	
**Non-essential amino acids**										
BGN variety	**Alanine**	**Glycine**	**Proline**	**Serine**	**Aspartic acid**	**Cysteine**	**Glutamic**	**Asparagine**	**Arginine**	**Tyrosine**
Cream Control	9.44 ± 1.73 ^a^	9.91 ± 1.95 ^a^	9.16 ± 1.75 ^a^	5.32 ± 0.35 ^a^	19.10 ± 0.45 ^a^	0.87 ± 0.04 ^b^	27.10 ± 0.29 ^a^	0.11 ± 0.00 ^a^	1.58 ± 0.30 ^a^	1.45 ± 0.01 ^a^
Dehulled	12.24 ± 2.26 ^b^	12.38 ± 2.03 ^b^	14.95 ± 3.17 ^b^	6.98 ± 1.00 ^c^	22.22 ± 0.66 ^c^	0.78 ± 0.03 ^a^	30.11 ± 0.16 ^b^	0.11 ± 0.01 ^a^	1.89 ± 0.21 ^a^	1.80 ± 0.22 ^b^
Malted	13.18 ± 2.58 ^b^	13.45 ± 2.86 ^b^	12.86 ± 2.11 ^b^	5.68 ± 0.08 ^b^	20.53 ± 0.67 ^b^	0.77 ± 0.01 ^a^	29.67 ± 0.13 ^b^	0.11 ± 0.03 ^a^	1.72 ± 0.26 ^a^	1.91 ± 0.54 ^b^
Brown Control	12.29 ± 0.33 ^a^	13.07 ± 1.45 ^a^	11.48 ± 0.78 ^a^	5.71 ± 0.21 ^a^	20.02 ± 0.23 ^a^	0.72 ± 0.00 ^a^	28.96 ± 0.54 ^a^	0.11 ± 0.01 ^a^	1.70 ± 0.16 ^a^	1.50 ± 0.08 ^a^
Dehulled	13.01 ± 0.70 ^b^	23.34 ± 2.58 ^c^	14.87 ± 0.85 ^b^	6.77 ± 0.37 ^b^	22.89 ± 0.32 ^b^	0.73 ± 0.01 ^a^	32.24 ± 0.83 ^b^	0.10 ± 0.01 ^a^	2.19 ± 0.29 ^b^	1.74 ± 0.06 ^b^
Malted	14.89 ± 1.90 ^b^	15.22 ± 1.58 ^b^	14.49 ± 0.25 ^b^	6.37 ± 0.04 ^ab^	22.36 ± 0.42 ^b^	0.87 ± 0.01 ^b^	30.19 ± 0.75 ^b^	0.11 ± 0.01 ^a^	2.36 ± 0.25 ^b^	1.72 ± 0.10 ^b^
Red Control	9.98 ± 0.04 ^a^	10.74 ± 0.51 ^a^	9.25 ± 1.06 ^a^	5.28 ± 0.72 ^a^	17.84 ± 0.67 ^a^	0.82 ± 0.02 ^a^	24.91 ± 1.05 ^a^	0.10 ± 0.00 ^a^	1.74 ± 0.36 ^a^	1.38 ± 0.13 ^a^
Dehulled	17.02 ± 1.39 ^c^	16.46 ± 0.96 ^b^	18.57 ± 2.95 ^c^	7.84 ± 0.87 ^b^	24.04 ± 0.79 ^c^	0.90 ± 0.02 ^b^	33.24 ± 1.82 ^c^	0.12 ± 0.02 ^a^	2.47 ± 0.63 ^b^	2.17 ± 0.71 ^b^
Malted	11.62 ± 0.30 ^b^	11.91 ± 0.57 ^a^	11.94 ± 0.25 ^b^	5.76 ± 0.26 ^a^	21.81 ± 0.46 ^b^	0.82 ± 0.00 ^a^	29.96 ± 1.19 ^b^	0.12 ± 0.02 ^a^	2.59 ± 0.25 ^b^	2.31 ± 0.23 ^b^
Total NEAA	114	126	118	56	191	7	236	1	18	16 = 883
TEAA:TNEAA ratio										0.41

The results are reported as the mean ± standard deviation of three replicates. Mean values in columns with different letters differ significantly (*p* ≤ 0.05). EAA = essential amino acids, NEAA = non-essential amino acids, T = total.

**Table 3:** Mineral content of control, dehulled and malted Bambara groundnut flours (%).

BGN Variety	Calcium	Phosphorus	Magnesium	Potassium	Sulfur	Zinc	Iron
Cream							
Control	0.97 ± 0.14 ^b^	51.52 ± 0.04 ^a^	2.53 ± 0.01 ^a^	0.54 ± 0.02 ^a^	51.47 ± 0.03 ^a^	0.97 ± 0.03 ^c^	0.63 ± 0.01 ^b^
Dehulled	0.94 ± 0.01 ^b^	52.48 ± 0.03 ^b^	2.99 ± 0.01 ^b^	0.64 ± 0.02 ^b^	52.48 ± 0.03 ^b^	0.94 ± 0.02 ^b^	0.54 ± 0.01 ^a^
Malted	0.82 ± 0.01 ^a^	55.82 ± 0.16 ^c^	4.65 ± 0.01 ^c^	1.02 ± 0.02 ^c^	55.87 ± 0.09 ^c^	0.82 ± 0.04 ^a^	0.81 ± 0.01 ^a^
Brown							
Control	0.88 ± 0.01 ^b^	50.30 ± 0.08 ^a^	3.38 ± 0.04 ^a^	0.82 ± 0.03 ^a^	50.08 ± 0.13 ^a^	0.98 ± 0.01 ^b^	0.81 ± 0.01 ^a^
Dehulled	0.77 ± 0.01 ^a^	52.19 ± 0.0 ^b^	3.61 ± 0.02 ^b^	1.12 ± 0.03 ^b^	52.23 ± 0.17 ^b^	0.77 ± 0.01 ^a^	0.71 ± 0.07 ^c^
Malted	0.77 ± 0.01 ^a^	54.02 ± 0.06 ^c^	4.42 ± 0.02 ^c^	1.44 ± 0.04 ^c^	54.08 ± 0.23 ^c^	0.82 ± 0.01 ^c^	1.12 ± 0.17 ^b^
Red							
Control	0.97 ± 0.01 ^c^	49.48 ± 0.03 ^a^	3.37 ± 0.02 ^b^	0.35 ± 0.01 ^a^	49.46 ± 0.13 ^a^	0.97 ± 0.01 ^b^	0.85 ± 0.09 ^c^
Dehulled	0.94 ± 0.01 ^b^	51.50 ± 0.03 ^b^	3.61 ± 0.03 ^c^	0.56 ± 0.02 ^b^	50.51 ± 0.02 ^b^	0.98 ± 0.01 ^b^	0.57 ± 0.10 ^a^
Malted	0.91 ± 0.01 ^a^	53.56 ± 0.25 ^c^	3.74 ± 0.03 ^a^	0.80 ± 0.04 ^c^	51.67 ± 0.10 ^c^	0.91 ± 0.01 ^a^	0.98 ± 0.11 ^b^

The results are reported as the mean ± standard deviation of three replicates. Mean values in columns with different letters differ significantly (*p* ≤ 0.05).

**Table 4:** Fatty acid composition of control, dehulled, and malted Bambara groundnut flours.

**Saturated Fatty Acids (µg/g)**							
**Sample**	**Myristic**	**Palmitic**	**Margaric**	**Stearic**	**Palmitoleic**	**Arachidic**	**Behenic**
Cream Control	5.42 ± 0.08 ^a^	235.19 ± 2.99 ^a^	2.88 ± 0.31 ^a^	78.35 ± 0.99 ^b^	2.05 ± 0.07 ^a^	23.01 ± 0.02 ^a^	72.96 ± 1.01 ^a^
Dehulled	5.39 ± 0.00 ^a^	242.61 ± 1.04 ^b^	2.92 ± 0.02 ^a^	76.38 ± 0.15 ^a^	2.04 ± 0.00 ^a^	23.41 ± 0.04 ^b^	74.10 ± 0.15 ^a^
Malted	5.39 ± 0.00 ^a^	242.61 ± 0.08 ^b^	2.93 ± 0.00 ^a^	75.95 ± 0.06 ^a^	2.03 ± 0.00 ^a^	23.47 ± 0.01 ^b^	74.01 ± 0.21 ^a^
Brown Control	5.40 ± 2.03 ^a^	243.99 ± 0.70 ^b^	2.95 ± 0.09 ^a^	75.42 ± 4.12 ^a^	2.12 ± 0.02 ^b^	24.83 ± 2.70 ^a^	72.17 ± 2.54 ^a^
Dehulled	5.40 ± 0.02 ^a^	242.34 ± 0.13 ^a^	2.94 ± 0.01 ^a^	76.49 ± 0.42 ^a^	2.05 ± 0.01 ^a^	23.78 ± 0.09 ^a^	73.79 ± 0.37 ^a^
Malted	5.39 ± 0.00 ^a^	242.51 ± 0.01 ^a^	2.93 ± 0.09 ^a^	76.10 ± 0.20 ^a^	2.04 ± 0.00 ^a^	23.53 ± 0.02 ^a^	73.98 ± 0.05 ^a^
Red Control	5.36 ± 1.03 ^a^	248.11 ± 2.31 ^b^	2.98 ± 0.26 ^b^	74.64 ± 2.31 ^a^	2.00 ± 1.00 ^a^	23.34 ± 1.51 ^a^	75.45 ± 6.86 ^a^
Dehulled	5.38 ± 0.01 ^a^	242.55 ± 2.55 ^a^	2.93 ± 0.03 ^a^	75.49 ± 0.49 ^a^	2.02 ± 0.01 ^a^	23.44 ± 0.11 ^a^	73.96 ± 0.69 ^a^
Malted	5.39 ± 0.00 ^a^	242.41 ± 0.21 ^a^	2.93 ± 0.10 ^a^	75.84 ± 0.00 ^a^	2.03 ± 0.10 ^a^	23.48 ± 0.00 ^a^	73.94 ± 0.05 ^a^
		**Unsaturated fatty acids (µg/g)**					
	**y-Linolenic**	**Elaidic**	**Linolelaidic**	**α- Linolenic**	**Eicosenoic**	**Docosadienoic**	**Dihomo-γ-linolenic acid**
Cream Control	23.59 ± 0.02 ^a^	272.10 ± 0.42 ^a^	440.46 ± 0.86 ^a^	27.77 ± 0.08 ^a^	10.21 ± 0.27 ^a^	20.33 ± 0.43 ^a^	4.20 ± 0.05 ^a^
Dehulled	23.99 ± 0.04 ^b^	280.13 ± 0.23 ^b^	444.52 ± 1.20 ^b^	29.36 ± 0.10 ^b^	10.61 ± 0.06 ^a^	20.70 ± 0.07 ^a^	4.27 ± 0.00 ^a^
Malted	24.05 ± 0.01 ^b^	280.35 ± 0.04 ^b^	443.48 ± 0.02 ^b^	29.52 ± 0.03 ^b^	10.62 ± 0.01 ^a^	20.65 ± 0.00 ^a^	4.26 ± 0.00 ^a^
Brown Control	21.42 ± 0.70 ^a^	278.89 ± 0.19 ^a^	442.68 ± 0.87 ^a^	31.28 ± 0.64 ^b^	10.66 ± 0.22 ^a^	20.49 ± 0.60 ^a^	4.02 ± 0.21 ^a^
Dehulled	24.38 ± 0.09 ^b^	280.02 ± 0.60 ^b^	443.43 ± 0.01 ^a^	29.72 ± 0.12 ^a^	10.59 ± 0.01 ^a^	20.64 ± 0.06 ^a^	4.22 ± 0.04 ^a^
Malted	24.12 ± 0.02 ^b^	280.28 ± 0.07 ^b^	443.48 ± 0.01 ^a^	29.55 ± 0.03 ^a^	10.61 ± 0.00 ^a^	20.65 ± 0.01 ^a^	4.25 ± 0.01 ^a^
Red Control	23.92 ± 0.08 ^a^	287.80 ± 2.45 ^b^	447.12 ± 0.39 ^b^	30.38 ± 2.69 ^a^	10.90 ± 0.98 ^a^	20.97 ± 2.52 ^a^	4.37 ± 0.36 ^a^
Dehulled	24.02 ± 0.11 ^b^	280.54 ± 2.98 ^a^	442.49 ± 3.58 ^a^	29.61 ± 0.29 ^a^	10.63 ± 0.10 ^a^	20.61 ± 0.23 ^a^	4.26 ± 0.04 ^a^
Malted	24.06 ± 0.06 ^b^	280.20 ± 0.27 ^a^	443.00 ± 0.22 ^a^	29.54 ± 0.03 ^a^	10.61 ± 0.01 ^a^	20.63 ± 0.02 ^a^	4.25 ± 0.00 ^a^

The results are reported as the mean ± standard deviation of three replicates. Mean values in columns with different letters differ significantly (*p* ≤ 0.05).

## Data Availability

The original contributions presented in this study are included in the article, and further inquiries can be directed to the corresponding author.
